# Supplementary material for: The Discovery of Ribosomal Protein bL31 from Escherichia coli: A Long Story Revisited
Source: Int J Mol Sci. 2023 Feb 8;24(4):3445. doi: 10.3390/ijms24043445 (PMC9966373; doi:10.3390/ijms24043445)
Supplement: Supplementary file 1 [file ijms-24-03445-s001.zip › ijms-2145538-supplementary.pdf]

SUPPORTING INFORMATION

Supporting References

1. Kaltschmidt, E.; Wittmann, H.G. Ribosomal proteins, XII. Number of proteins in small and large subunits of *Escherichia coli* as determined by two-dimensional gel electrophoresis. *Proc. Natl. Acad. Sci. USA* **1970**, 67, 1276–1282. https://doi: 10.1073/pnas.67.3.1276. PMID: 4922286

2. Agirrezabala, X.; Liao, H. Y.; Schreiner, E.; Fu, J.; Ortiz-Meoz, R. F.; Schulten, K.; Green, R.; Frank, J. Structural characterization of mRNA-tRNA translocation intermediates. *Proc. Natl. Acad. Sci. USA*, **2012,** 109, 6094–6099. https://doi.org/10.1073/pnas.12012 88109. PMID: 22467828

Supporting Figures

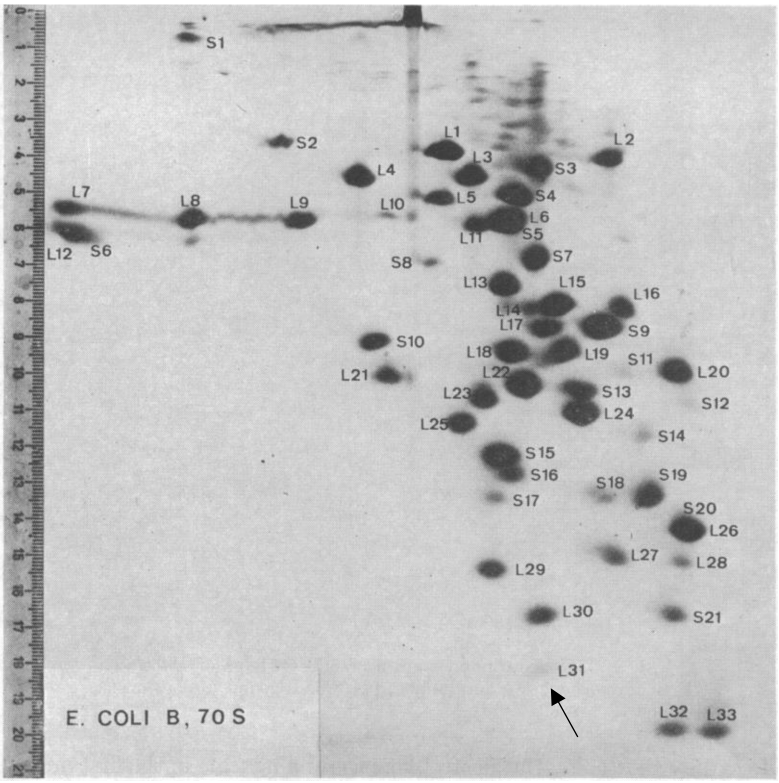

Figure S1. 2D electrophoretogram of 70S r-proteins of *E. coli*. Electrophoresis in the first dimension used 4% acrylamide and pH 8.6. Electrophoresis in the second dimension used 18% acrylamide and pH 4.6. In the first dimension, the anode was on the left. In the second dimension, the anode was at the top **[1]**. The arrow indicates L31. In Figure S1, "b" of r-proteins has been omitted.

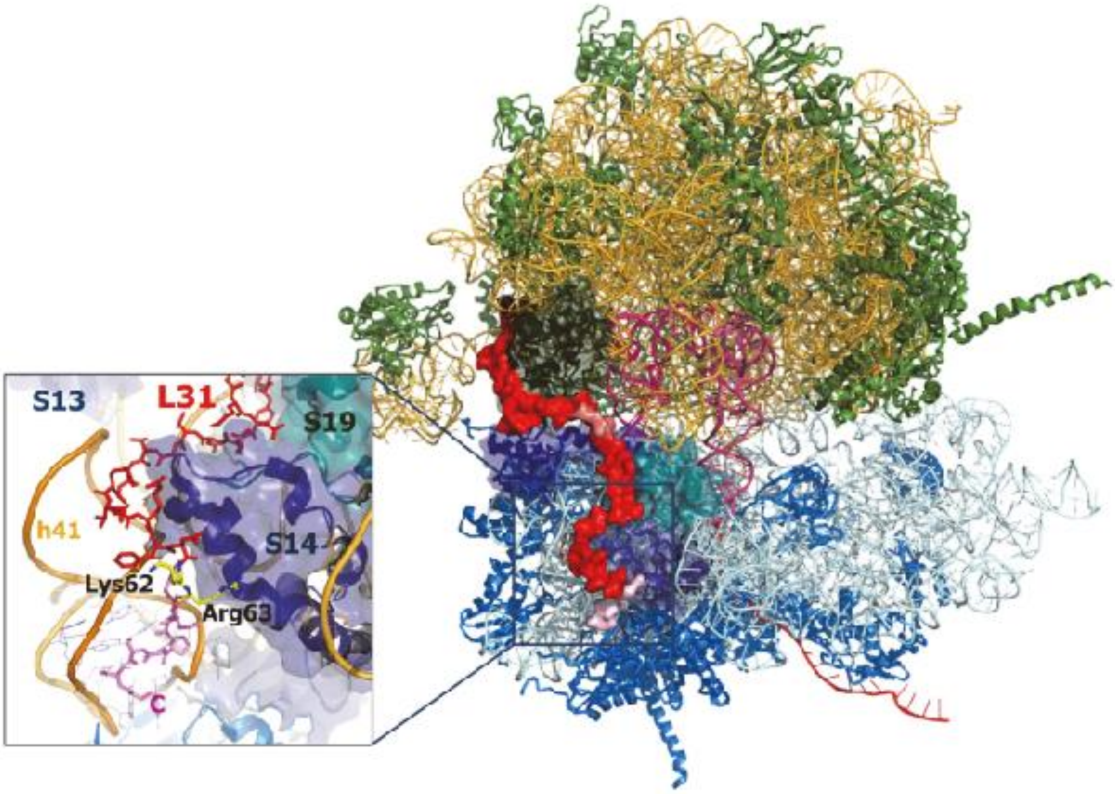

Figure S2. The structural location of *E. coli* bL31 on the 70S ribosome. The 50S r-proteins (green), 30S r-proteins (blue), 23S and 5S rRNAs (gold), 16S rRNA (gray), mRNA (reddish brown), and A- and P-site tRNAs (magenta) are indicated in the ribosomal structure. bL31 protein is represented as a surface model (on the whole ribosome) and a stick model (on the close-up window), with the N-domain (Met1–Leu32) and C-domain (Val36-Lys62) colored red and the connective peptide (Asn33-Asp35) and C-tail (Arg63-Lys70**)** cleaved by protease 7 colored pink. In the close-up window, Lys62, Arg63, and the C-terminus of bL31 are indicated. bS13, bS19, and bS14 are indicated as ribbon models. Helix-41 (h41) is colored orange, with partial line models for the nucleotides that bind to the C-tail of bL31. The model was generated using cryo-electron microscopy structures of the *E. coli* ribosome (PDB code 4V6N) **[2]**. In Figure S2, "b" of r-proteins has been omitted.

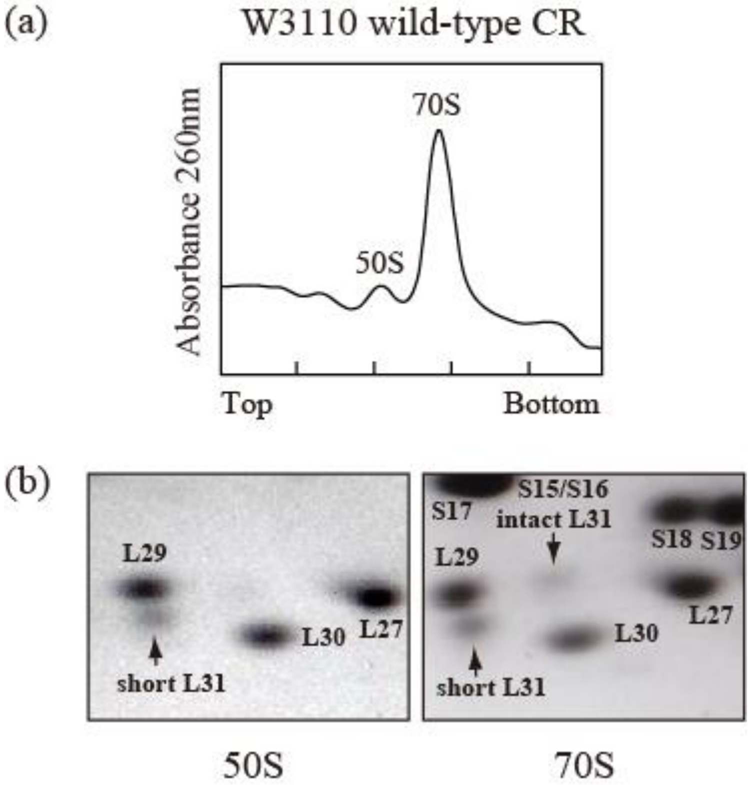

Figure S3. The 70S fraction contains intact and short bL31, but the 50S fraction contains only short bL31. CRs from W3110 cells, which were grown in EP medium at 37 °C and collected in exponential growth phase (Klett units: ~ 50), were prepared using buffer I [20 mM Tris-HCl (pH 7.6), 15 mM magnesium acetate, 100 mM ammonium acetate, and 6 mM 2-mercaptoethanol] and separated into 50S and 70S by 10–40% preparative SDG centrifugation using buffer I. Each fraction of 50S and 70S was collected, and proteins were analyzed by RFHR 2D PAGE. (a) The ribosome profile after 5–20% analytical SDG centrifugation is shown. (b) r-proteins in the 50S or 70S fractions are shown with enlarged images of intact bL31, short bL31, and the surrounding area. In Figure S3, "b" of r-proteins has been omitted.

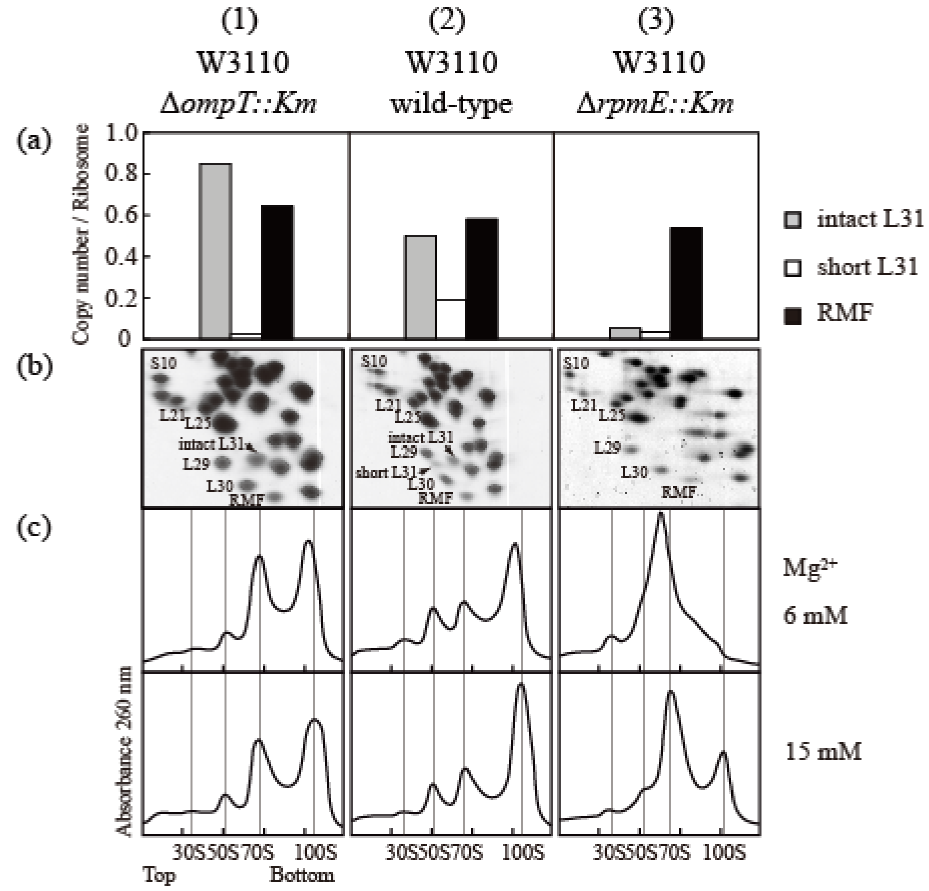

Figure S4. Ribosomes lacking bL31 are defective in 100S formation in stationary phase. (1) W3110 *ΔompT::Km*, (2) wild-type, and (3) *ΔrpmE::Km* cells were grown at 37 °C for 3 days in EP medium and harvested. CRs were prepared and analyzed by RFHR 2D PAGE and 5–20% SDG centrifugation in the presence of 6 or 15 mM Mg^{2+}. (a) Copy numbers per ribosome of intact bL31 (gray), short bL31 (white), and RMF (black) in each CR sample are shown by bars. Copy numbers were calculated from gel spots shown in (b). (b) Panels show the pattern of CRs resolved on RFHR 2D PAGE gels. Spots corresponding to bS10, bL21, bL25, bL29, bL30, intact bL31, short bL31, and RMF are indicated. (c) Ribosome profiles after 5–20% SDG centrifugation in the presence of 6 or 15 mM Mg^{2+}. For each CR sample, 150 pmol was used. In Figure S4, "b" of r-proteins has been omitted.

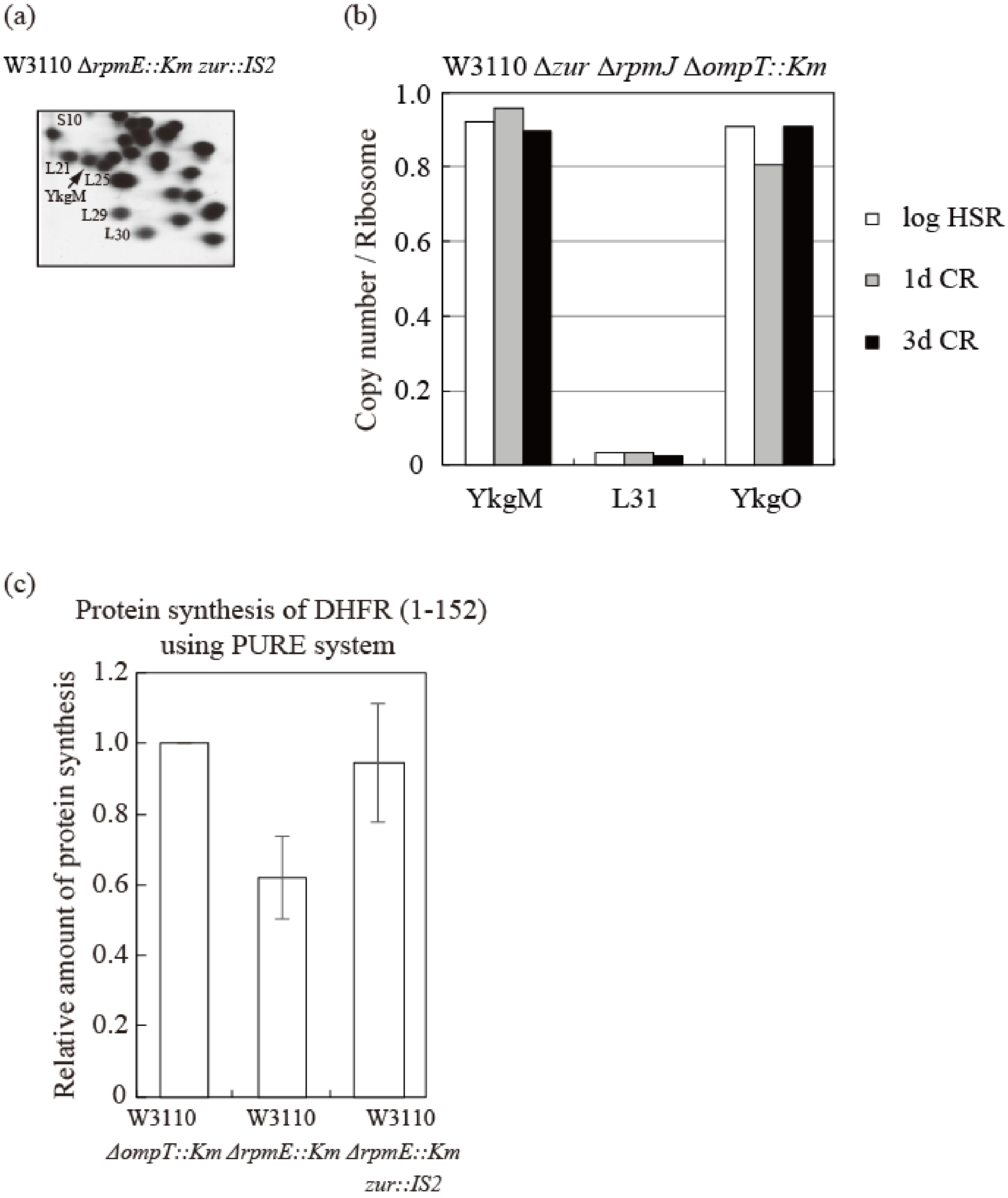

Figure S5. Ribosomes prepared from W3110 *ΔrpmE::Km zur::IS2* cells contain one copy of YkgM. (a) W3110 *ΔrpmE::Km zur::IS2* cells were grown at 37 °C in EP medium and collected in logarithmic growth phase (Klett units: 50). HSRs were prepared. HSR proteins were analyzed by RFHR 2D PAGE. Gels were stained with CBB. Spots corresponding to r-proteins bS10, bL21, bL25, bL29, bL30, and YkgM are indicated. (b) Ribosomes of W3110 *Δzur ΔrpmJ ΔompT::Km* cells contain one copy of YkgM. W3110 *Δzur ΔrpmJ ΔompT::Km* mutant cells were grown in EP medium at 37 °C and collected in logarithmic growth phase (Klett units: 50) or stationary phase

(1 and 3 days). HSRs (for logarithmic phase) or CRs (for stationary phase) were prepared, and r-proteins were analyzed by RFHR 2D PAGE. The copy numbers of bL31, YkgM, and YkgO in HSRs or CRs were quantified by RFHR 2D PAGE. The vertical axis shows the copy number per ribosome. Copy numbers of bL31, YkgM, and YkgO in ribosomes prepared in logarithmic phase (Klett units: 50) (white) or cells cultured for 1 (gray) or 3 (black) days are shown. (c) In vitro translational activity of ribosomes lacking bL31 is 40% lower than that of ribosomes containing intact bL31, but this activity is almost completely recovered in ribosomes containing YkgM. Synthesis of DHFR (1–152) was measured using a purified in vitro transcription-translation system. HSRs prepared from three strains (W3110 *ΔompT::Km*, *ΔrpmE::Km*, and *ΔrpmE::Km zur::IS2*) were added to the purified system. After electrophoresis of reaction mixtures on 10–20% linear gradient SDS PAGE gels, synthesized DHFR (1–152) was visualized by CBB staining. The density of the DHFR band was normalized against that of the specific r-protein band. Protein synthesis was normalized against synthesis by HSRs containing only intact bL31 (i.e., *ΔompT::Km*). The data are averages of four independent reactions and two PAGE gels per reaction (a total of eight gels). In Figure S5, "b" of r-proteins has been omitted.

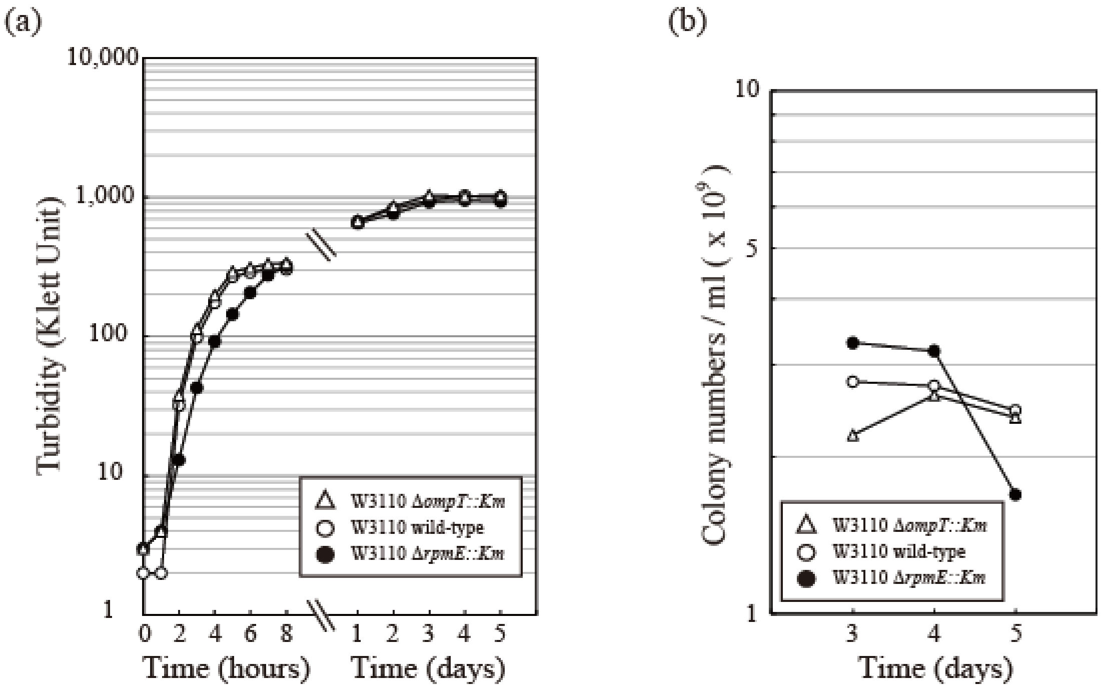

Figure S6. Depletion of bL31 inhibits bacterial growth in liquid EP medium. (a) W3110 *ΔompT::Km* (open triangles), W3110 wild-type (open circles), and W3110 *ΔrpmE::Km* (closed circles) strains were cultured in EP medium at 37 °C. Cell growth was monitored by measuring Klett units. The time scales are 0–8 h (left side) and 1–5 days (right side). The vertical axis shows normal logarithmic values. (b) Colony-forming unit (CFU) values of the three strains listed in (a). Cultured cells were harvested after 3, 4, or 5 days of cultivation, and the CFU value was measured.
